# Artemisinin resistance-associated markers in *Plasmodium falciparum* parasites from the China-Myanmar border: predicted structural stability of K13 propeller variants detected in a low-prevalence area

**DOI:** 10.1371/journal.pone.0213686

**Published:** 2019-03-18

**Authors:** Yan He, Susana Campino, Ernest Diez Benavente, David C. Warhurst, Khalid B. Beshir, Inke Lubis, Ana Rita Gomes, Jun Feng, Wang Jiazhi, Xiaodong Sun, Fang Huang, Lin-hua Tang, Colin J. Sutherland, Taane G. Clark

**Affiliations:** 1 National Institute of Parasitic Diseases, Chinese Center for Disease Control and Prevention, Shanghai, Shanghai, People’s Republic of China; 2 WHO Collaborating Centre for Malaria, Schistosomiasis and Filariasis, Key Laboratory of Parasite and Vector Biology, Ministry of Health, Shanghai, People’s Republic of China; 3 Faculty of Infectious and Tropical Diseases, London School of Hygiene and Tropical Medicine, London, United Kingdom; 4 Yunnan Institute of Parasitic Diseases, Provincial Centre of Malaria Research, Provincial Collaborative Innovation Centre for Public Health and Disease Prevention and Control, Provincial Key Laboratory of Vector-borne Diseases Control and Research, Puer, China; 5 Tengchong County Centers for Disease Control and Prevention, Guanghua village, Tiancheng district, Tengchong, Yunnan Province, China; 6 Faculty of Epidemiology and Population Health, London School of Hygiene and Tropical Medicine, London, United Kingdom; Instituto Rene Rachou, BRAZIL

## Abstract

**Background:**

Malaria reduction and future elimination in China is made more difficult by the importation of cases from neighboring endemic countries, particularly Myanmar, Laos, and Vietnam, and increased travel to Africa by Chinese nationals. The increasing prevalence of artemisinin resistant parasites across Southeast Asia highlights the importance of monitoring the parasite importation into China. Artemisinin resistance in the Mekong region is associated with variants of genes encoding the K13 kelch domain protein (*pf13k*), found in specific genetic backgrounds, including certain alleles of genes encoding the chloroquine resistance transporter (*pfcrt*) and multidrug resistance transporter PgH1 (*pfmdr1*).

**Methods:**

In this study we investigated the prevalence of drug resistance markers in 72 *P*. *falciparum* samples from uncomplicated malaria infections in Tengchong and Yingjiang, counties on the Yunnan-Myanmar border. Variants of *pf13k*, *pfcrt* and *pfmdr1* are described.

**Results:**

Almost all parasites harboured chloroquine-resistant alleles of *pfcrt*, whereas *pfmdr1* was more diverse. Major mutations in the K13 propeller domain associated with artemisinin resistance in the Mekong region (C580Y, R539T and Y493H) were absent, but F446I and two previously undescribed mutations (V603E and V454I) were identified. Protein structural modelling was carried out *in silico* on each of these K13 variants, based on recently published crystal structures for the K13 propeller domain. Whereas F446I was predicted to elicit a moderate destabilisation of the propeller structure, the V603E substitution is likely to lead to relatively high protein instability. We plotted these stability estimates, and those for all previously described variants, against published values for *in vivo* parasitaemia half-life, and found that quadratic regression generates a useful predictive algorithm.

**Conclusion:**

This study provides a baseline of *P*. *falciparum* resistance-associated mutations prevalent at the China-Myanmar border. We also show that protein modelling can be used to generate testable predictions as to the impact of *pfk13* mutations on *in vivo* (and potentially *in vitro*) artemisinin susceptibility.

## Introduction

In China, the use of qinghao (*Artemisia annua* L.) for alleviating febrile illnesses has been traced back to 168 BC, but it was not until the 1970’s that purified artemisinin was shown to inhibit malaria parasites, especially in drug-resistant strains [1]. Artemisinin combination therapy (ACT) is now used worldwide as the first line treatment for falciparum malaria. This highly successful approach is now under threat as a significant delay in parasite clearance after artemisinin monotherapy has become prevalent in the greater Mekong sub region (GMS), in Southeast Asia [2, 3]. In a major breakthrough for surveillance and malaria control efforts, mutations in the *Pfk13 gene* (*PF3D7_1343700*) have been identified to be strongly associated with reduced susceptibility to artemisinin in the GMS, both *in vitro* and in the field [4]. Unlike alleles associated with chloroquine (CQ)-resistance, which spread from Southeast Asia to Africa in the 1970s, the markers associated with reduced susceptibility to artemisinin have not been observed in sub-Saharan Africa. Several mutations were identified that can be useful for surveillance, including three high-frequency allele changes (C580Y, R539T and Y493H) that are strongly associated with extended parasite clearance times *in vivo*, and enhanced survival after a pulse of 700nM dihydroartemisinin (DHA) *in vitro* [5]. In Myanmar and southern China these variants of major concern are not found, and instead the F446I mutation dominates; this is associated with a moderately prolonged parasite clearance half-life [6, 7] but not with therapeutic failure after treatment with ACT [8]. Other genes may play a role in modulating artemisinin or partner drug susceptibility. Particular alleles of *pfcrt* and *pfmdr1* are associated with artemisinin and ACT failure in the Mekong, but these variants have not been evaluated on the Yunnan–Myanmar border [9, 10]. Specific alleles of *pfcrt* and *pfmdr1* are also known to be selected for by ACT treatment in Kenya and Uganda [11, 12].

The artemisinin drug family has a history of over three decades of use in China, first as monotherapy, then combined with partner compounds. Integrated into the national malaria surveillance-response system established in 2004, artemisinin has assisted the country to significantly reduce malaria disease burden and is supporting the aim to reach elimination [13]. A major problem for malaria reduction in China is the importation of cases from other countries. This is particularly challenging for Yunnan, where locally-acquired malaria is nearing elimination, as the province shares borders with relatively high-burden malaria endemic countries including Myanmar, Laos, and Vietnam. The spread of artemisinin-resistant parasites across Southeast Asia, and the presence of an endemic malaria transmission zone in Yunnan renders very important the monitoring of *Pfk13* alleles and other markers of drug resistance in *P*. *falciparum* from malaria cases presenting in that region.

In this study we investigated the prevalence of polymorphisms in *pfk13*, *pfcrt* and *pfmdr1* in seventy-two *P*. *falciparum* isolates from individuals with uncomplicated malaria presenting between 2012 to 2015 in Tengchong and Yingjiang, two counties at the Yunnan-Myanmar border. We also explored the potential functional impact of the observed *pfk13* variants by estimating impacts on propeller domain stability predicted by structural modelling. These disruptive effect estimates were in turn modelled against published data on parasitaemia half-life *in vivo* for known variants, to develop predictive algorithms of the effects on parasite clearance half-life for the six *Pfk13* mutations identified in our sample set.

## Materials and methods

### Sample collection, PCR and DNA sequencing

Dried blood filter paper samples were collected from eighty outpatients, confirmed positive for *P*. *falciparum* infection by microscopy at clinics in Tengchong and Yingjiang districts, Yunnan, China from 2012 to 2015. *P*. *falciparum* isolate DNA was extracted from the filter papers using the Chelex100 method described elsewhere [14], with 72 (90%) samples providing sufficient DNA for marker sequencing. These samples were characterized for *Pfk13* and *pfmdr1* mutations using nested PCR amplification and capillary sequencing [11], as well as for *pfcrt* codons (72–76) using probe-based qPCR as previously described [15, 16]. The K13-propeller gene fragment (coordinates 1726169–1726997 on chromosome 13; gene ID PF3D7_1343700) was amplified by nested PCR using standard primers [5] as previously described [17]. The sequence of amplified DNA products was determined using ABI PRISM 3730 Genetic Analyser (Applied Biosystems, UK). Chromas software (Technelysium, Australia) was used to analyse the sequence results. The sequence data were complemented by SNPs characterized from other populations (18 countries, n = 2,000), which have been described previously [18, 19], using an established bioinformatics pipeline [20, 21]. For ease of interpretation, the samples were grouped into geographical regions. In addition, two recent studies with *pfk13* mutation data for Chinese samples were used for comparisons ([22], n = 329; [5], n = 2).

### Protein structural modelling

The effects of mutations identified in *pfk13*, were estimated by assessing the effects of the residue substitution on a 3-dimensional protein structure. The Delta-delta Gibbs energy value (ΔΔG, kcal.mol^-1^) of folding was calculated assuming the wild type has a ΔΔG of zero and that variant K13 sequences have destabilising negative (-ΔΔG) or stabilizing positive (+ΔΔG) values. The analysis was carried out on the computation suites DUET [23] and SDM2 [24], and used the unlinked (4YY8b) and S-S linked (4ZGCa) unaltered crystal structures of K13 directly downloaded by DUET and SDM2 from the RCSB database [25, 26]. Using post-dosing parasitaemia median half-life data from at least 24 patients for each gene variant [5, 22, 27], we fitted a regression model with the “resistance” outcome against the ΔΔG values for the present mutations. This model allowed predictions of *in vivo* clearance half-life from the disruptive –ΔΔG values calculated. Predictions of *in vivo* parasite clearance half-life for K13 variants mapped onto either the 4YY8b or 4ZGCa structures were obtained by inclusion of ΔΔG values as a linear or quadratic effect in the regression model. The goodness of fit was determined by estimating the Robust Standard Deviation of the Residuals (RSDR). The GraphPad Prism and the R statistical packages were used to analyse the results.

### Ethical considerations

The study was reviewed and approved by the ethical review committees of the National Institute of Parasitic Diseases, Chinese Center for Disease Control and Prevention (China CDC), and of the WHO Western Pacific Regional Office. Informed written consent was obtained from adult patients and guardians of minor patients.

## Results

### *Pfcrt* and *pfmdr1* genes

A total of 72 uncomplicated *P*. *falciparum* infected samples were collected from Yingjiang and Tengchong Counties, Yunnan Province, and the provincial Parasitic Disease Institute from 2012 to 2015 (**S1 Table**). The patients were residents in Yunnan and acquired *P*. *falciparum* infection locally (n = 8), regionally (Myanmar; n = 53) or overseas (Africa; n = 11). Sixty-six isolates were sequenced successfully for *pfcrt* and 32 isolates for *pfmdr1*. *Pfcrt* CQ-resistant haplotypes (CVIET or SVMNT at codons 72–76) were observed in 100%, 98% and 36.6% of the parasites from China, Myanmar and Africa, respectively, consistent with recent reported patterns [18]. Thus the majority of *P*. *falciparum* infections were predicted to be chloroquine and/or amodiaquine resistant (62/69, 89.9%). Five of the cases harbouring the CQ-sensitive *pfcrt* haplotype CVMNK included 4 “self-reported” from Africa (Cameroon, Chad, Mali, and Nigeria), and one from Myanmar. In four patients we detected multiple *pfcrt* genotypes with both CVMNK and CVIET haplotypes occurring together (Mali, Nigeria and Cameroon). The CVIET haplotype was the most frequent allele in our study, consistent with multiple populations in Southeast Asia and Africa (**Table 1**). The SVMNT haplotype was only present in Myanmar in years 2014 and 2015 (**Table 1**).

**Table 1:** Mutations in *Pfcrt* and *Pfmdr1*.

Gene	Resistance Mutation / haplotype	Freq.***	Source**	Observed elsewhere*
*crt*	CVMNK	0.15	Myanmar, Africa	Bangladesh (7%), Burkina Faso (41%), Cambodia Northeast (3.7%), DRC (25%), Gambia (27%), Ghana (70%), Guinea (25%), Kenya (60%), Laos (10%), Mali (26%), Malawi (100%), Tanzania (22%), Vietnam (6%)
	CVIET	0.95	Myanmar, Africa, China	Bangladesh (93%), Burkina Faso (56%), Cambodia North (70%), Cambodia Northeast (55%), Cambodia West (95%), Colombia (6%), DRC (77%), Gambia (73%), Ghana (29%), Guinea (74%), Kenya (35%), Laos (60%), Mali (63%), Myanmar Central (100%), Myanmar South (100%), Nigeria (100%), Peru (42%), Tanzania (72%), Thailand East (94%), Thailand South (100%), Thailand West (99%), Vietnam (60%)
	SVMNT	0.08	Myanmar	Peru (57%), PNG (100%)
*mdr1*	N86Y	0.15	Myanmar, Africa	Bangladesh (25%), Burkina Faso (36%), Cambodia West (0.5%), DRC (53%), Gambia (27%), Ghana (25%), Guinea (46%), Kenya (53%), Mali (45%), Malawi (3%), Nigeria (25%), Peru (14%), PNG (90%), Tanzania (66%), Thailand South (5%), Vietnam (0.5%)
	D90H	0.05	China	-
	V104A	0.05	Africa	-
	Y184F	0.50	Myanmar, Africa, China	Bangladesh(18%), Burkina Faso (77%), Cambodia North(40%), Cambodia Northeast (4.7%), Cambodia West (81%), Colombia (100%), DRC (51%), Gambia (69%), Ghana (76%), Guinea (68%), Kenya (13%), Laos (3%), Mali (74%), Malawi (60%), Myanmar Central (39%), Myanmar South (40%), Nigeria (75%), Peru (86%), PNG (9%), Tanzania (38%), Thailand East (88%), Thailand South (50%), Thailand West (15%), Vietnam (27%)
	S1034I	0.05	Myanmar	West Thailand (0.6%)
	F1226Y	0.15	Myanmar, China	West Thailand (60%), Myanmar South (28.9%), Myanmar Central (24.5%), Vietnam (7.4%), Cambodia North (3%), Cambodia Northeast (3.7%), Cambodia West (0.3%), Laos (0.3%)

* From [18]

** participant self-reported; DRC Democratic Republic of Congo

*** there are mixed infections, leading to the total *crt h*aplotype frequencies being >1

Six point mutations in *pfmdr1* were identified across 16 of 32 samples sequenced. These chloroquine-resistance associated mutations included the known N86Y (n = 3), Y184F (n = 10) and S1034I (n = 1) and the novel D90H (n = 2), V104A (n = 1) and F1226Y (n = 3) observed in both local and imported cases. The Y184F mutation was the most frequent (31.4%) and was found in indigenous, Myanmar and African sourced infections. This observation is consistent with its observed high frequency in Southeast Asian and African populations (**Table 1**). Double mutants were also detected in 4 isolates, including N86Y\D90H (n = 2, Myanmar and indigenous), N86Y\Y184F (n = 1, Myanmar) and V104A\Y184F (n = 1, Mali). Whilst, N86Y has been observed more often in African populations, it has been seen in low frequency in Southeast Asia (**Table 1**). The V104A (Mali) and S1034I (Myanmar) mutations were only detected in single cases, and unobserved in the larger dataset (**Table 1**). There are two samples with both *pfcrt* K76T and *pfmdr1* N86Y mutations.

### *Pfk13* gene

**Fig 1:**
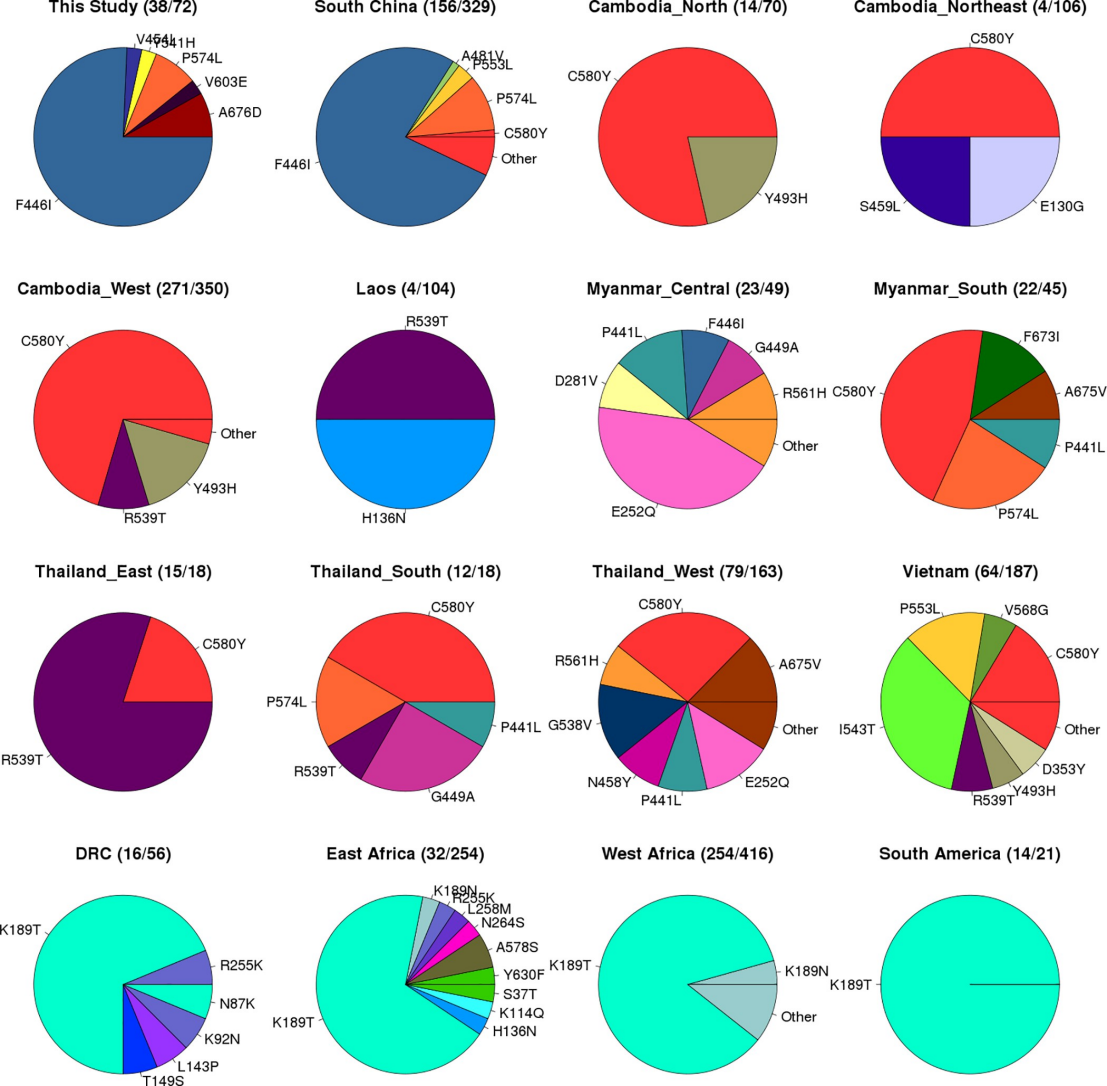
Frequency of *PfK13* mutations*. * (number resistance / number of samples in brackets), all data from [18], except our study and South China [22]; DRC Democratic Republic of Congo.

*Pfk13* mutations were detected in half the samples (38/72), with 6 different nonsynonymous mutations characterized (**Table 2**). These mutations included the previously reported F446I (n = 28, 38.9%), P574L (n = 3), A676D (n = 3) and Y541H (n = 1), and two novel mutations V603E (n = 1) and V454I (n = 1) (**Table 1, Fig 1**). The F446I mutation occurred at the highest frequency (39.6% Myanmar, 18.2% Africa (possible Asian acquisition), 62.5% China) and was the only K13 propeller domain mutation observed in individuals who recently came back from Africa. This mutation has been observed across Thailand-Myanmar-China frontier (~20.2%) [5], and in a Southern Chinese population (44.0%) [22], but not in Cambodia-Vietnam-Lao PDR [5] and other Southeast Asian or African populations (**Fig 1**). Interestingly, none of the individuals harbouring the *pfk13* F446I mutation in Myanmar or China also harboured the SVMNT *pfcrt* haplotype, but rather carried the CVIET genotype. The observed presence of the P574L K13 variant at low frequency is supported by observations from other studies involving Chinese populations ([5, 22]; **Fig 1**). We identified the A676D mutation in Myanmar and China, the first reports in Southeast Asia, although this variant was previously described in a Guinean population (**Table 2**). The two novel mutations (V603E, V454I) detected here each occurred in only one sample (China or Myanmar), and no previous reports of either were found in the literature. The artemisinin susceptibility-associated C580Y, R539T, Y493H and I543T variants found in Cambodia-Vietnam-Lao PDR [5, 27] and other populations in the Mekong region, were absent in our data; an observation consistent with other studies in Southern China and bordering Myanmar (**Fig 1**). The absence of P553L, a mutation previously found in Chinese populations, was not unexpected as its frequency has been reported as low (<1%) [22]. Similarly, N458Y and R561H mutations found in Thailand-Myanmar-China (both ~2% frequency; [5]) were not detected among our samples.

**Table 2:** Mutations identified in *PF3D7_1343700*.

Codon	K13 Propeller Blade	DUET ΔΔG 4YY8b/4ZGCa	SDM2 ΔΔG 4YY8b/4ZGCa	Mut. Freq.	Our study Source***	Observed elsewhere**
F446I	I	-1.51/-1.30	0.23/0.23	0.375	Myanmar, China, Africa	Myanmar Central (6.1%), Thailand West (1.2%); Thailand-Myanmar-China (20.2%) *
V454I	I	0.001/-0.08	-0.03/-0.03	0.014	Myanmar	-
Y541H	III	-2.18/-2.25	-1.42/-1.46	0.014	China	-
P574L	III-IV	-0.52/-0.45	-0.58/-0.58	0.042	Myanmar	Myanmar South (11.1%), Thailand South (16.6%), Vietnam (1.0%); Thailand-Myanmar-China (3.1%) *
V603E	IV	-2.81/-2.66	-1.62/-1.62	0.014	Myanmar	-
A676D	VI	-0.85/-0.95	-0.22/-0.82	0.042	Myanmar, China	Guinea (2.1%)

ΔΔG = Delta-delta Gibbs energy, where highly negative values result in protein instability

** data from [18]

unless

* = [5]

*** participant self-reported; note, the common C580Y, R539T, Y493H, and I543T mutations were not present in our samples.

### Protein structural modelling

**Fig 2:**
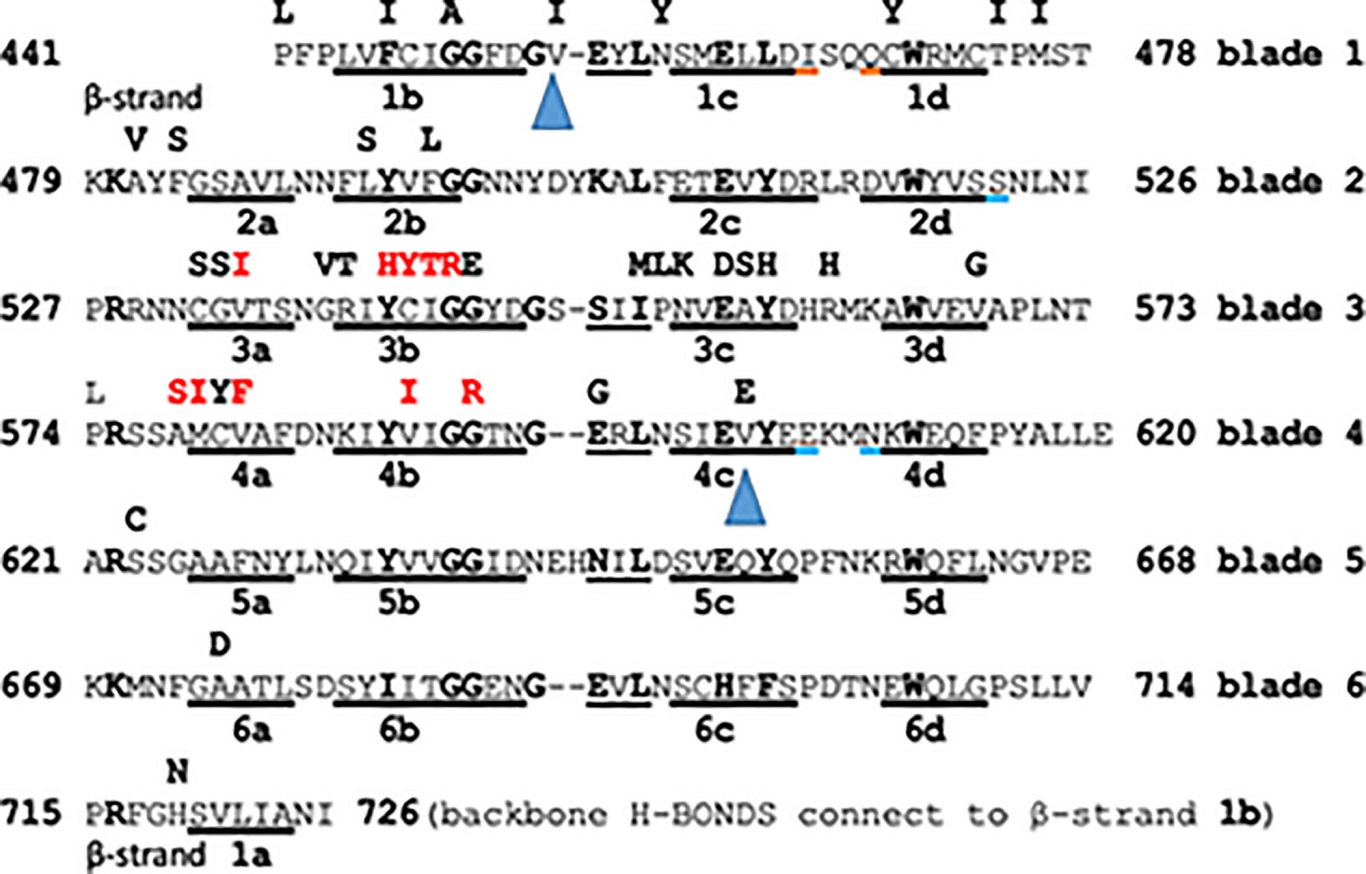
Sequence alignment for the *Pfk13* propeller using 4YY8b and S-S 4ZGCa models. Alignment of the 6 Kelch propeller blades of K13 as predicted by *in silico* modelling (structures RCSB 4YY8b and 4ZGCa with a Cys532-Cys580 link; [25, 26]); inter-blade residue similarities are emphasised in bold. Each of the β-strands in each blade is underlined and presented in order of the sequence. After the 6^th^ blade, the terminal β-strand 1a binds at the start to complete the propeller structure before β-strand 1b. The characteristic kelch markers L-W, Y-W, Y-W, Y-W, Y-W and F-W occur in each blade and each span 6 intervening residues. Individual residue replacements are seen in bold above each blade. Ten variant residues depicted in red were seen to promote the Cys 532-Cys 580 link in 4YY8b and prevent it in 4ZGCa, when tested using the SDM (2011) suite. In Cys-Cys–linked 4ZGCa the illustrated β-strand includes the brown-underlined residues, and excludes the blue-underlined residues. In all predicted structures, the basic Arg or Lys residues in position 2 of each row contribute their protonated side chain N atom(s) to form a ring of charge around the lower entrance of the propeller channel. Each of the 2 mutations V454I and V603E is underlined by a triangular blue marker.

To explore the effects of the observed mutations, including V603E and V454I, we estimated the protein stability ΔΔG based on alignments for both the 4YY8b and di-sulphide linked 4ZGCa crystal structures (**Fig 2;** [25, 26]; **S1 Fig**) using the DUET and SDM2 servers (**S2 Table**). A quadratic model of the relationship between ΔΔG value and observed drug half-life (in hours) is presented (**S2 Fig)**. Most of the important residue changes which impact on the parasitaemia half-life in patients are seen in the β-strands of the Pfk13 propeller structure (**S2 Table**), and this is not unexpected in view of the rather important role of β-strand to β-strand H-bonding in the rigidity of the propeller. There are, however residue changes from wild type which are located in loops between the β-strands, such as the A578S variant.

We considered parasitaemia half-life predictions based on mutations identified in our study. The V454I loop mutation between β-strands 1b and 1c had only a small protein disruption effect on (DUET ΔΔG: 4YY8 0.001; 4ZGC -0.08), and the predicted half-life (4YY8 3.36; 4ZGC 3.99 hours) was close to the observed wild-type (median 3.32; China only 3.17 hours). However, the inter- β-strand loop P574L polymorphism shows greater disruption (ΔΔG: 4YY8–0.52; 4ZGC -0.45), with predicted half-lives of 4.89 and 5.19 (median of 5.17 hours was observed for the 7 patients). The importance of this loop disruption is emphasised by the fact that the residue following P574 is positively-charged Arg575, which together with 5 other basic residues, Arg622, Lys670, Arg716, Lys480 and Arg528 forms a structural ring around the inner channel of the propeller, important in stabilizing the whole structure. The apparently novel V603E and known Y541H mutations are expected to cause more disruptive effects on protein structure (DUET: ΔΔG < -2.25), leading to a prolongation of the predicted *in vivo* parasite half-life (>7.5 hours), greater than the estimates for the highly frequent F446I mutation (median half-life 5.89; DUET predicted < 7.15). A676D has an intermediate disruptive effect (DUET ΔΔG = -0.85, -0.95) and half-life (5.67, 6.48) (**S2 Fig**), similar to known C580Y and R539T effects in other populations. In field studies, an observed post-artemisinin parasite clearance half-life greater than 5 hours is considered to be an indicator of resistance [27]. The SDM2 modelling results were broadly similar to those from DUET **(Table 2; S2 Table; S2 Fig**). However, there were two notable discrepancies (R539T, F446I), which SDM2 predicted were stabilising mutations (ΔΔG values >0) and led to additional quadratic estimation (**S2 Fig)**. Although the resulting clearance half-lives were similar to those from DUET, this situation highlights that there is further scope for improvement in stability estimation from protein structural models, and more broadly, that any results should be validated using phenotypic assays and larger sample collections.

## Discussion

The objective to eliminate malaria in the China-Myanmar border region is threatened by the emergence of artemisinin resistance [28]. Knowledge of mutations in the *k13-propeller (Pfk13)* gene associated with slow clearance of artemisinin derivatives, provides the ability to track and prevent spread, and assess the effectiveness of control measures. Here we examined 72 *P*. *falciparum* malaria infected patients, all Chinese residents, living in Yunnan, with known recent travel history. Chloroquine was withdrawn as a treatment in the China-Myanmar border area almost four decades ago [29, 30]. However, nearly all parasite strains from this region still retain resistant forms of *pfcrt*, with no evidence of recovery of sensitivity to chloroquine, as previously reported in other countries [31, 32]. For *pfcrt* 72–76 codons, the “CVIET” resistance haplotype is dominant, occupying 85.5%. We also investigated the presence of mutation in the *Pfmdr1* gene that has been implicated in modulating the response to artemisinin and ACT [11, 12, 33, 34]. The amino-acid mutations N86Y and Y184F are the most common reported mutations identified worldwide and were also detected in this study in Asian and African parasites. Three novel *pfmdr1* mutations were also detected at low frequency (D90H, V104A and F1226Y).

Six mutations in the *Pfk13* gene were identified, including F446I and P574L, which are known border-regional surveillance markers [13]. The F446I mutation has the highest frequency, which is consistent with previous reports [35, 36]. Surprisingly, two cases apparently imported from Cameroon and Nigeria were detected with the F446I mutation. This mutation was not thought to be present in sub-Saharan Africa [37], and local acquisition cannot be ruled out in these patients. The phenotype of F446I and P574L variants remains to be established, but our modelling predicts that the former has disruptive effects on the protein and would potentially lead to slower clearance of parasites. Interestingly, F446I has never previously been found in parasites harbouring the SVMNT haplotype in *pfcrt*, and in the available global collection the F446I is always found in parasites of the CVIET genotype. This is an important observation, as the SVMNT haplotype was previously very common throughout the Mekong region, but all K13-mediated artemisinin resistance cases described to date have occurred in the presence of the CVIET haplotype of *pfcrt*, accompanied by specific variants of *pfcrt* that are newly emerging under ACT pressure, particularly that of the partner drug piperaquine [10]. Any interaction among these loci should be explored in larger prospectively collected datasets.

Other known border region K13 variants, such as N458Y and R561H, as well as others prevalent in regions further east (C580Y, R539T, Y493H, I543T) were not detected in this study. Two of the polymorphisms identified here at low frequency, V603E and V454I, are apparently novel. V454I has minimal predicted effects on the protein structure, being close to wild-type estimates. However, V603E is expected to be highly disruptive of propeller domain stability, leading to a predicted parasite clearance half-life greater than that of C580Y, R539T, and Y493H, and close to I543T. It is very likely however, that the greater degrees of protein disruption (-) or stabilization (+) will start to affect parasite fitness as ΔΔG values decline below –2.0 or rise to above 2.0. Phenotypic studies of parasites carrying these variants would be most instructive. In all predicted structures, we note a “ring” of positively charged side-chains at the lower entrance of the propeller channel (**Fig 2**). An orthologous set of six residues in the human kelch propeller domain protein Keap1 bind with high-affinity negatively-charged residues in the N-terminal portion NEH2 of the oxidative-stress regulatory peptide Nrf2 before its ubiquitination and final destruction by the proteome [38]. In spite of the likely distinct function of the plasmodial propeller protein these important features are still retained. Interestingly there is, among *P*. *falciparum* genomic products a WD40 repeat protein, Pf 3D7 W7K5T2, which retains practically the full sequence for the human NRF2 high-affinity site for Kelch 1 propeller (see Clustal alignment in **S1 Fig**).

The case of the A578S K13 variant is particularly interesting as, according to the –ΔΔG value, it is predicted to have a large disruptive effect, and has been associated with slow parasite clearance in a small group of Ugandan malaria patients [39] but not in a single Kenyan patient [17]. Further, this variant did not reduce susceptibility of the CQ-resistant line Dd2 *in vitro*, following gene-edited residue change [5]. As A578S is most prevalent in Africa, where parasites are more likely to carry wild-type *pfcrt*, genetic background may be an important consideration when trying to harmonise findings from these different studies, and more extensive work is needed to determine the true impact of this K13 variant.

There are some potential limitations to our work. First, we did not consider all relevant resistance markers, but future surveillance work will assess *plasmepsin* 2 and *pfmdr1* gene copy number, and sequence variants of *pfap2mu* and *pfubp1* in our study area. Second, the sample size is small, but the frequencies of the observed mutations are in keeping with elsewhere, and our study provides a baseline for ongoing surveillance. Third, there is some uncertainty in the protein structural modelling, but across the models and software tools implemented, the results are similar. It is helpful that we have been able to use the original crystal structures submitted to the RCSB database [25, 26]. Fourth, it is possible that the relationship between the disruptive effects of mutations and parasitemia half-life (surrogate for parasite clearance) may be confounded by host genetics, including the patient G6PD and haemoglobin variants (e.g. HbE) in South East Asia, which varies in different regions [40], as well as underlying *pfcrt* and *pfmdr1* genotypes. The quadratic relationship fits the observed clearance data and provides a starting point for inferring the effects of novel *Pfk13* mutations. As more data become available this model will improve.

Overall, our approach provides additional detail of *P*. *falciparum* resistance gene mutations in the China-Myanmar region, thereby assisting elimination efforts. We also utilise a novel modelling framework to make predictions as to the effects of uncharacterised “resistance” mutations on the ability of artemisinin-derived drugs to rapidly clear parasites, increasing the utility of DNA sequence data for inference of phenotype in settings where patient follow-up or *ex vivo* parasite culture is not possible.

## Conclusions

Malaria control and prevention in the China-Myanmar region has focused on case-based interventions. This region has unique characteristics in part due to drug use history, geography and movement of migrants. Our work has shown the utility of genetics in identifying resistance gene variants in a surveillance setting, and provided further evidence of parasite polymorphisms associated with slow clearance of *P*. *falciparum* after artemisinin derivative treatment, which can inform current malaria elimination efforts.

## Supporting information

S1 Table Study samples. (PDF)

S2 Table Comparison between observed and predicted median half-life (MHL) of parasitaemia using linear and quadratic (ΔΔG) analysis of PfK13 mutations with the –ΔΔG values, based on analysis of crystal structures 4YY8b and S-S linked 4ZGCa using the DUET website. (PDF)

S1 Fig CLUSTAL Omega (version 1.2.4) multiple sequence alignment of the low-affinity and high affinity sites of human NRF2 (bold) which bind to the Keap 1 propeller protein and proposed similar sites in the *P. falciparum* WD40 repeat protein. (PDF)

S2 Fig Quadratic relationship between parasitaemia half-life and inferred Delta-delta Gibbs energy (DDG, ΔΔG). Predictions (solid squares) for V603E, Y541H, A578S, A676D, P574L, and V454I are presented (blue); WT = wild type; horizontal dashed lines are the medians per mutation.
(PDF)
