# Supplementary material for: Artemisinin resistance-associated markers in Plasmodium falciparum parasites from the China-Myanmar border: predicted structural stability of K13 propeller variants detected in a low-prevalence area
Source: PLoS One. 2019 Mar 18;14(3):e0213686. doi: 10.1371/journal.pone.0213686 (PMC6422288; doi:10.1371/journal.pone.0213686)
Supplement: S1 Table — (PDF) [file pone.0213686.s001.pdf]

S1 Table

Study samples

**Source**	**2012**	**2013**	**2014**	**2015**	**Total**
Indigenous	2	2	4	-	8
Myanmar	14	4	21	14	53
Africa	-	-	8	3	11*
Total	16	6	33	17	72

* includes travellers to Cameroon (n=2), Chad (n=1), Ethiopia (n=1), Mali (n=3), and Nigeria (n=4)
