# Supplementary material for: Artemisinin resistance-associated markers in Plasmodium falciparum parasites from the China-Myanmar border: predicted structural stability of K13 propeller variants detected in a low-prevalence area
Source: PLoS One. 2019 Mar 18;14(3):e0213686. doi: 10.1371/journal.pone.0213686 (PMC6422288; doi:10.1371/journal.pone.0213686)
Supplement: S2 Table — (PDF) [file pone.0213686.s002.pdf]

S2 Table

Comparison between observed and predicted median half-life (MHL) of parasitaemia using linear and quadratic (∆∆G) analysis of *PfK13* mutations with the –∆∆G values, based on analysis of crystal structures 4YY8b and S-S linked 4ZGCa using the DUET website.

Although the linear analysis gave a P value <0.0001, the quadratic analysis was more predictive (F-statistic $P<10^{-9}$), with predictions closer to the observed half-lives, and values for unlinked 4YY8 having marginal improvements over those from S-S linked 4ZGC.

Model	Mutant	Obs. MHL	DUET ∆∆G	Linear MHL*	Quad. MHL*	SDM2 ∆∆G	Linear MHL*	Quad. MHL*	Structure	Patients
4YY8b	WT	3.17	0.00	5.10	3.36	0.00	5.04	3.23/3.18		43 China
	F446I	5.89	-1.51	6.45	6.87	0.23	6.15	5.95	B-STRAND 1b	49 China
	V454I		0.001	5.11	3.36	-0.03	5.07	3.34	LOOP 1b-1c	
	V493H	7.40	-2.28	7.14	7.58	-0.97	6.02	6.11	B-STRAND 2b	24
	R539T	5.80	-1.08	6.06	6.14	0.21	5.92	5.68	B-STRAND 3b	45
	Y541H		-2.18	7.04	7.53	-1.42	6.48	6.99	B-STRAND 3b	
	I543T	7.56	-3.43	8.14	7.29	-3.29	8.38	7.54	B-STRAND 3b	30
	P574L	5.17	-0.52	5.56	4.87	-0.58	5.63	5.11	LOOP 3d-4a	7 China
	A578S		-1.45	6.39	6.77	-1.66	6.72	7.34	LOOP 3d-4a	
	C580Y	6.50	-1.06	6.04	6.10	-1.15	6.20	6.49	B-STRAND 4a	264
	V603E		-2.81	7.59	7.64	-1.62	6.68	7.29	B-STRAND 4c	
	A676D		-0.85	5.86	5.67	-0.22	5.26	3.99	B-STRAND 6a	
4ZGCa	WT	3.17	0.000	5.52	3.69	0.000	5.77	3.93/3.18		43 China
	F446I	5.89	-1.30	6.39	7.15	0.23	6.10	5.95	B-STRAND 1b	49 China
	V454I		-0.08	5.58	3.98	-0.03	5.79	4.08	LOOP 1b-1c	
	Y493H	7.40	-2.35	7.10	7.99	-1.46	6.71	8.74	B-STRAND 2b	24
	R539T	5.80	-0.71	6.00	5.91	0.22	5.98	5.68	B-STRAND 3b	45
	Y541H		-2.25	7.04	7.98	-1.46	6.71	8.74	B-STRAND 3b	
	I543T	7.56	-3.54	7.90	6.84	-3.29	7.88	7.30	B-STRAND 3b	30
	P574L	5.17	-0.45	5.83	5.19	-0.58	6.14	6.47	LOOP 3d-4a	7 China
	A578S		-1.47	6.51	7.40	-1.75	6.89	9.07	LOOP 3d-4a	
	C580Y	6.50	-0.74	6.02	5.99	-0.47	6.07	6.05	B-STRAND 4a	264
	V603E		-2.66	7.31	7.90	-1.62	6.81	8.95	B-STRAND 4c	
	A676D		-0.95	6.16	6.48	-0.82	6.30	7.28	B-STRAND 6a	

* Predicted
