# Supplementary material for: Artemisinin resistance-associated markers in Plasmodium falciparum parasites from the China-Myanmar border: predicted structural stability of K13 propeller variants detected in a low-prevalence area
Source: PLoS One. 2019 Mar 18;14(3):e0213686. doi: 10.1371/journal.pone.0213686 (PMC6422288; doi:10.1371/journal.pone.0213686)
Supplement: S1 Fig — (PDF) [file pone.0213686.s003.pdf]

S1 Figure

CLUSTAL Omega (version 1.2.4) multiple sequence alignment of the low-affinity and high affinity sites of human NRF2 (bold) which bind to the Keap 1 propeller protein and proposed similar sites in the *P. falciparum* WD40 repeat protein.

```
NRF2          QQDMDLIDILWRQDIDLGVSR-EVFDFSQR----------------RKEYELEKQKKL--  54
PfW7K5T2      DKGLNSFIILWKINETSFLDPIKKFYYNCEENEKKRTHIYNNKKKNQDEYDINQINDNTI 177
              ::.:: : ***: :    :.  : * :. .                 :.**::::  :.

NRF2          EK--ERQE---------QLQKEQ----------EKA-FFAQLQLDEETGE 82
PfW7K5T2      DNSSENGDAINNNVGYECIDISHDNRYICALTEEKIYIKSNTKYDDETGE 227
              ::  *. :          :: .:          **  : :: : *:****
```

In NRF2 there are seven lysine **(K)** residues between the 2 sites, six of which are destined to be ubiquitinated in the propeller. In the *P. falciparum* WD40 repeat protein there are six Lys residues between the putative low and high affinity sites. A closely similar picture for putative low and high affinity sites in WD40 was confirmed in *P. reichenowi* and *P. gaboni*, although sufficient sequence was not available in the PlasmoDB database to confirm the similarity of the proposed high affinity binding site in other species.
