# Supplementary material for: Artemisinin resistance-associated markers in Plasmodium falciparum parasites from the China-Myanmar border: predicted structural stability of K13 propeller variants detected in a low-prevalence area
Source: PLoS One. 2019 Mar 18;14(3):e0213686. doi: 10.1371/journal.pone.0213686 (PMC6422288; doi:10.1371/journal.pone.0213686)
Supplement: S2 Fig — Predictions (solid squares) for V603E, Y541H, A578S, A676D, P574L, and V454I are presented (blue); WT = wild type; horizontal dashed lines are the medians per mutation. (PDF) [file pone.0213686.s004.pdf]

S2 Figure

Quadratic relationship between parasitaemia half-life and inferred Delta-delta Gibbs energy (DDG, ΔΔG). Predictions (solid squares) for V603E, Y541H, A578S, A676D, P574L, and V454I are presented (blue); WT = wild type; horizontal dashed lines are the medians per mutation

(a) 4YY8b (DUET)

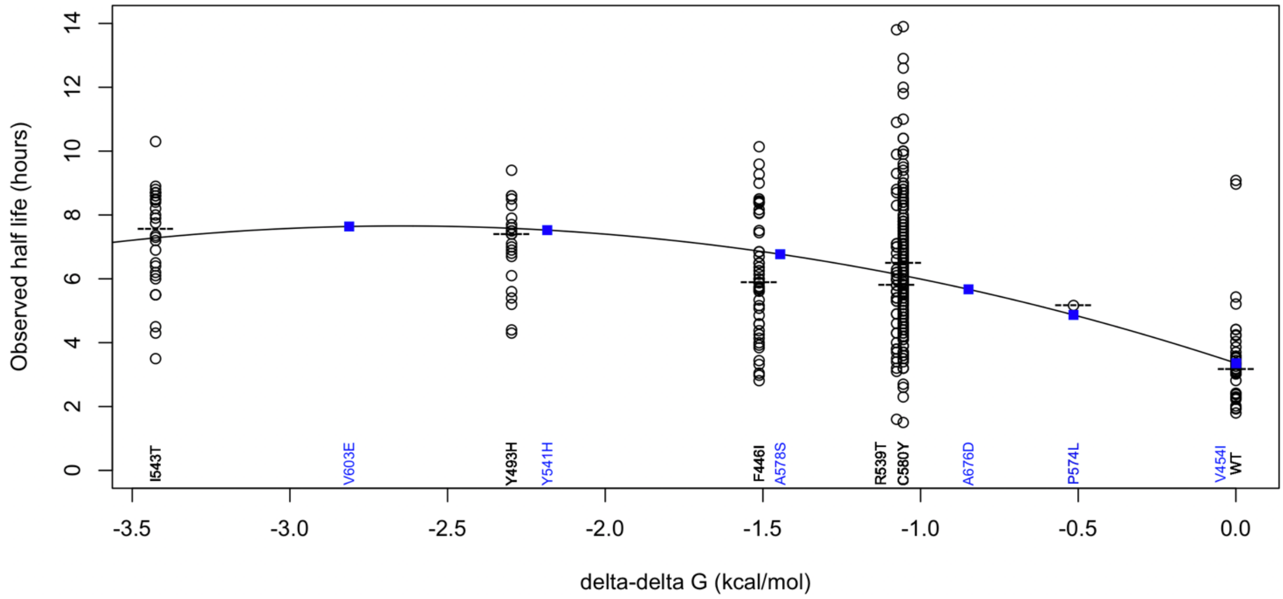

Black line: 3.359 – 3.248 DDG - 0.614 DDG^2; adjusted R^2 = 0.174; inclusion of quadratic effect ($P<6x10^{-10}$)

(b) 4ZGCa (DUET)

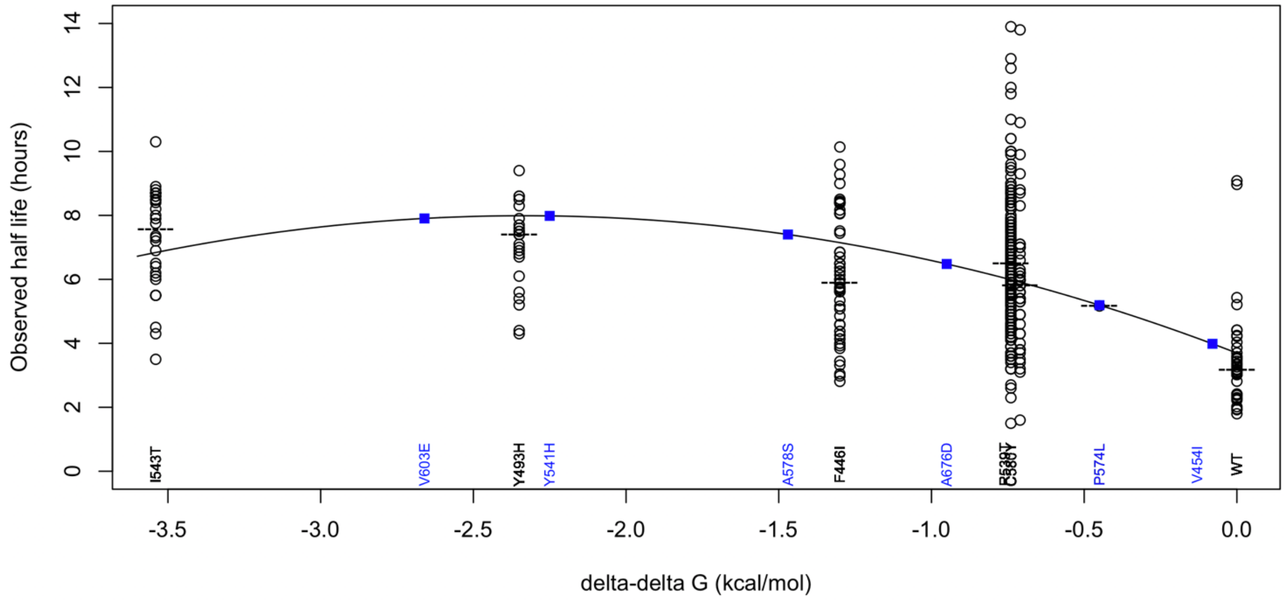

Black line: 3.696 – 3.681 DDG - 0.789 DDG^2; adjusted R^2 = 0.140; inclusion of quadratic effect ($P<3x10^{-9}$)

(c) 4YY8b (SDM2)

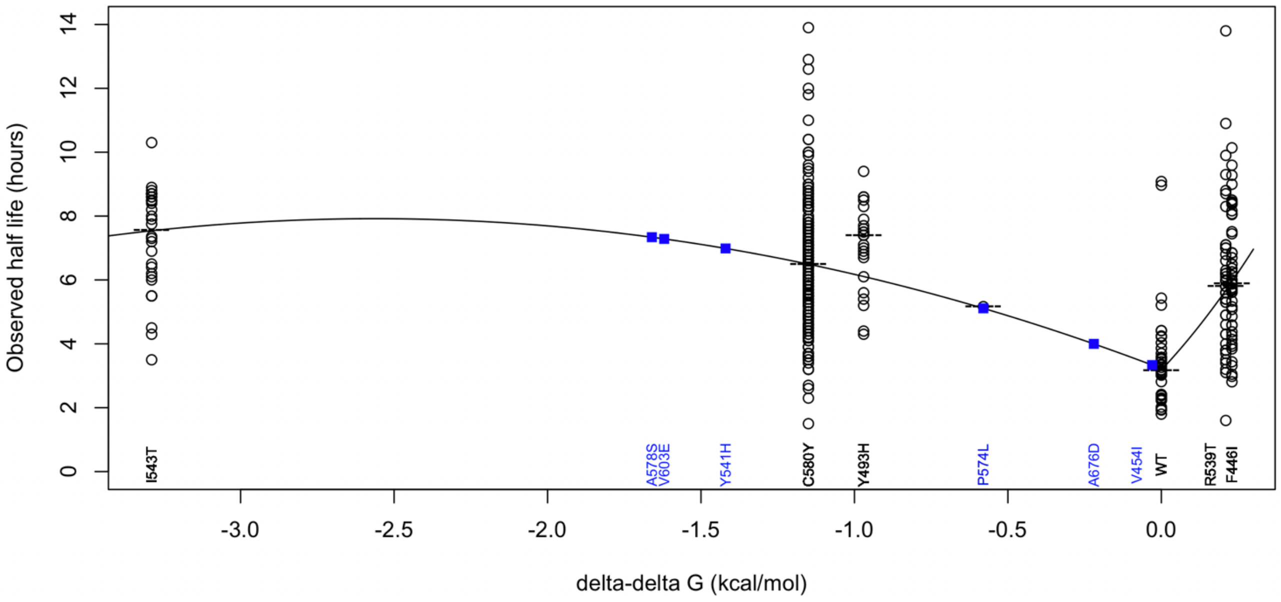

Black lines: (negative DDG) 3.227 – 3.663 DDG - 0.715 DDG^2; (positive DDG) 3.182 + 10.18 DDG + 8.087 DDG^2; adjusted R^2 = 0.246; inclusion of quadratic effect ($P<7x10^{-13}$)

(d) 4ZGCa (SDM2)

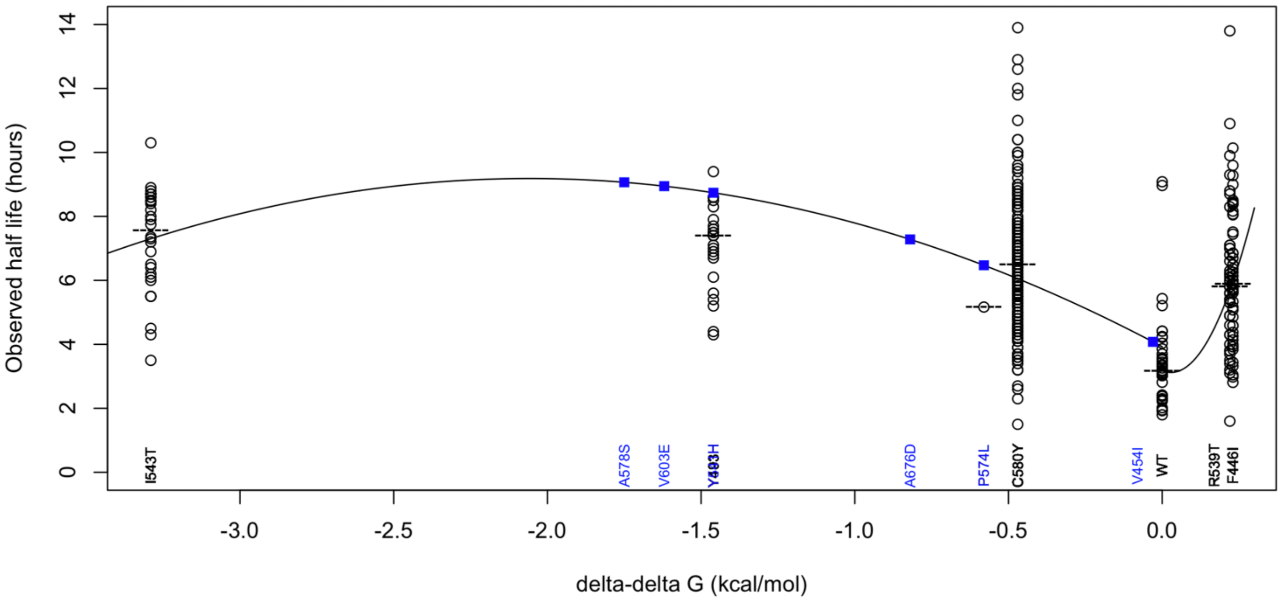

Black lines: (negative DDG) 3.926 – 5.109 DDG – 1.241 DDG^2; (positive DDG) 3.182 - 4.096 DDG + 70.15 DDG^2; adjusted R^2 = 0.185; inclusion of quadratic effect ($P<2x10^{-12}$)
